# Supplementary material for: Antimicrobial and Antibiofilm Potential of Thymus vulgaris and Cymbopogon flexuosus Essential Oils against Pure and Mixed Cultures of Foodborne Bacteria
Source: Antibiotics (Basel). 2023 Mar 13;12(3):565. doi: 10.3390/antibiotics12030565 (PMC10044171; doi:10.3390/antibiotics12030565)
Supplement: Supplementary file 1 [file antibiotics-12-00565-s001.zip › antibiotics-2199478-supplementary.pdf]

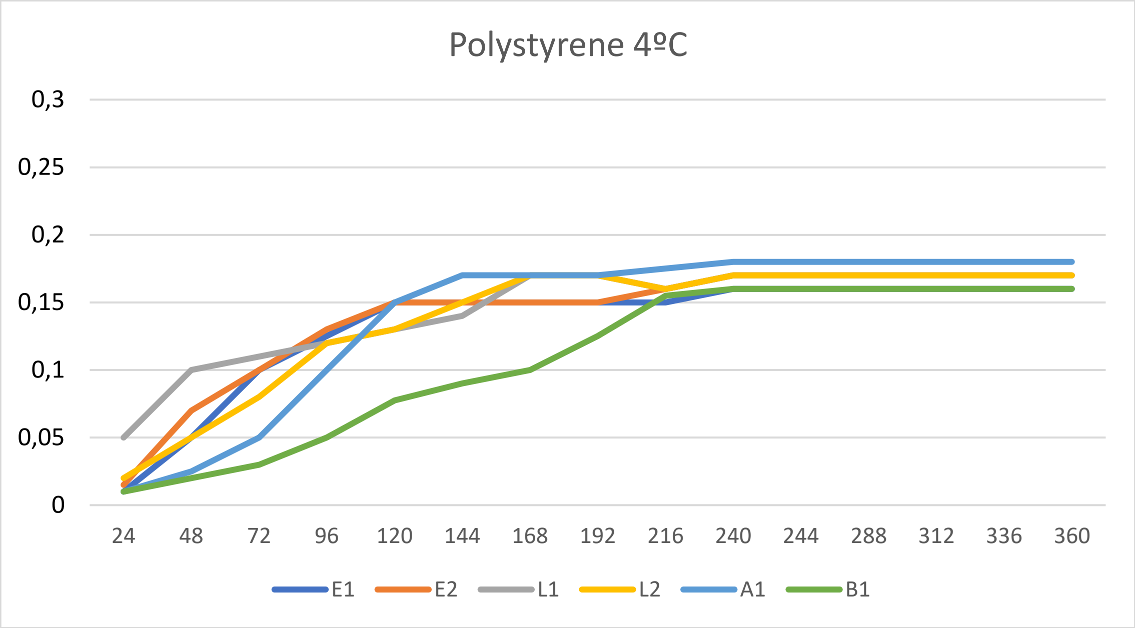

Figure S1- Biofilm biomass over-time (Horizontal axis: hours; vertical axis: optical density. Average from triplicate assays).

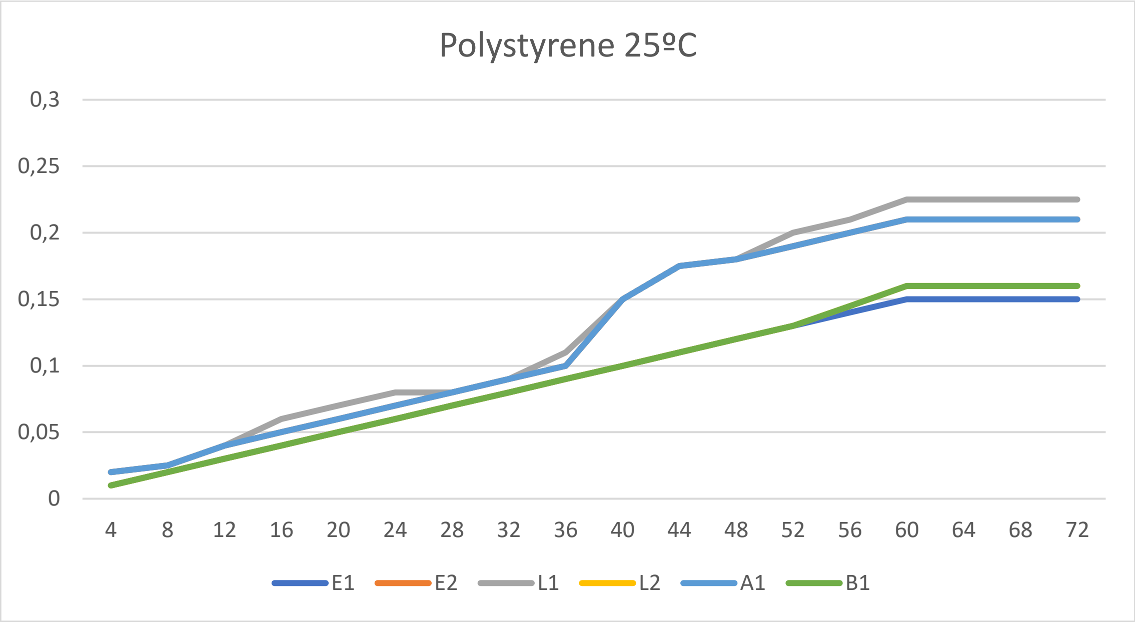

Figure S2- Biofilm biomass over-time (Horizontal axis: hours; vertical axis: optical density. Average from triplicate assays). Note: E2 results coincided with E1.

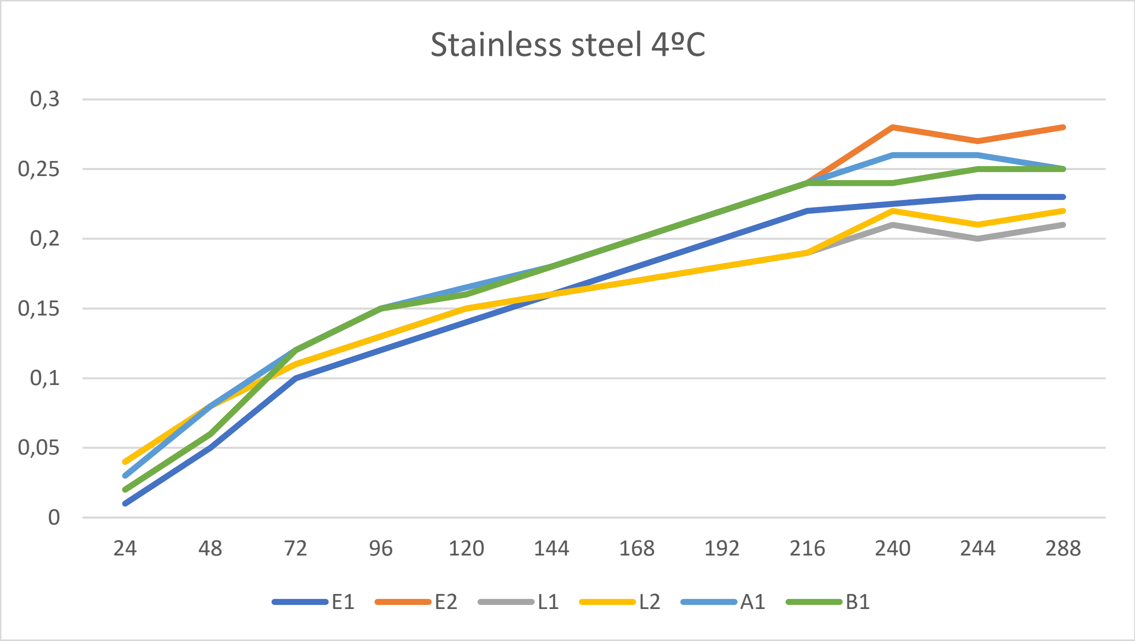

Figure S3- Biofilm biomass over-time (Horizontal axis: hours; vertical axis: optical density. Average from triplicate assays).

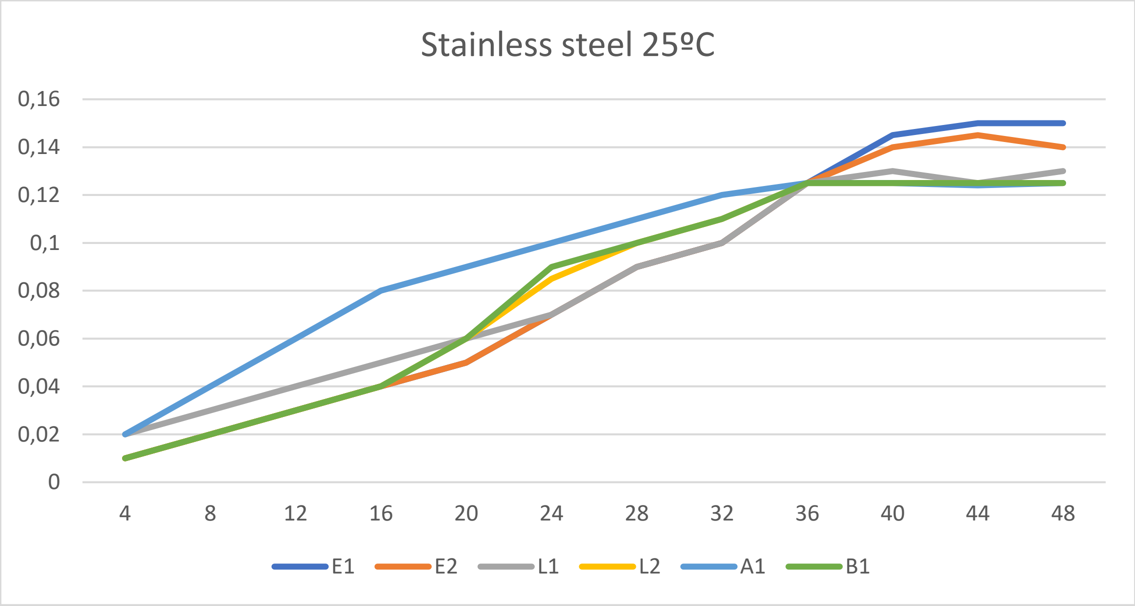

Figure S4- Biofilm biomass over-time (Horizontal axis: hours; vertical axis: optical density. Average from triplicate assays).
